# Peripheral inflammatory pain sensitisation is independent of mast cell activation in male mice

**DOI:** 10.1097/j.pain.0000000000000917

**Published:** 2017-04-05

**Authors:** Douglas M. Lopes, Franziska Denk, Kim I. Chisholm, Tesha Suddason, Camille Durrieux, Matthew Thakur, Clive Gentry, Stephen B. McMahon

**Affiliations:** Wolfson Centre for Age-Related Diseases, King's College London, United Kingdom

**Keywords:** Mast cell, Pain, Inflammation, NGF, Neuroimmunology

## Abstract

The immune and sensory systems are known for their close proximity and interaction. Indeed, in a variety of pain states, a myriad of different immune cells are activated and recruited, playing a key role in neuronal sensitisation. During inflammatory pain it is thought that mast cells (MC) are one of the immune cell types involved in this process, but so far the evidence outlining their direct effect on neuronal cells remains unclear. To clarify whether MC are involved in inflammatory pain states, we used a transgenic mouse line (Mctp5Cre-iDTR) in which MC could be depleted in an inducible manner by administration of diphtheria toxin. Our results show that ablation of MC in male mice did not result in any change in mechanical and thermal hypersensitivity in the CFA model of inflammatory pain. Similarly, edema and temperature triggered by CFA inflammation at the injection site remained identical in MC depleted mice compared with their littermate controls. In addition, we show that Mctp5Cre-iDTR mice display normal levels of mechanical hypersensitivity after local injection of nerve growth factor (NGF), a factor well characterised to produce peripheral sensitisation and for being upregulated upon injury and inflammation. We also demonstrate that NGF treatment in vitro does not lead to an increased level of tumor necrosis factor-α in bone marrow-derived MC. Furthermore, our qRT-PCR data reveal that MC express negligible levels of NGF receptors, thereby explaining the lack of response to NGF. Together, our data suggest that MC do not play a direct role in peripheral sensitisation during inflammatory conditions.

## Abstract 2

Supplemental Digital Content is Available in the Text.

Mast cells do not play a role as a direct nociceptor sensitiser during acute inflammatory pain and in mechanical hypersensitivity triggered by local nerve growth factor.

## 1. Introduction

The cross-talk between sensory and immune systems is well recognised. Indeed, the close physical proximity of nociceptors and immune cells, particularly in peripheral tissues, makes these systems key subjects of study in both acute and chronic pain states.^34,53,68,71^ Though the mechanisms of interaction are not fully understood, a number of studies demonstrate that on injury, inflammation triggers the activation of resident and innate immune cells. This results in the release of proinflammatory mediators, culminating in the sensitization of nociceptors, hyperalgesia, and persistent pain.^53,68,83^ Growing evidence also suggests that nociceptors in turn can impact on immune cell function, modulating intracellular properties and giving rise to some long lasting conditions, such as arthritis.^1,19,24,40,53^

Whereas, some populations of immune cells, such as macrophages and neutrophils, have an established role in neuropathic and inflammatory pain,^7,8,20,48,56,64,68,76,85^ the role of mast cells (MC) as a mediator of nociceptor sensitisation is much less clear. Mast cells are known for the presence of granule-like structures (vesicles), which on activation release a variety of inflammatory mediators, including chemokines, growth factors, and neuropeptides.^2,29,37,60^ Beyond their well-established role in allergy due to histamine secretion, MC have also been linked to an array of chronic pathological conditions such as bladder pain syndrome, irritable bowel syndrome, and migraine.^2,5,16,46,60,61^ Furthermore, a few studies suggest that MC might be involved in acute peripheral inflammatory pain. It has been shown that thermal and mechanical hypersensitivity resulting from systemic administration of nerve growth factor (NGF) could be prevented if MC function was blocked.^47^ Since then, other studies using different inflammatory models have also proposed a role for MC in hyperalgesia and allodynia.^23,25,69,81,85^ Most recently, it was shown that mice with disrupted c-Kit signalling (a kinase crucial for MC development^10^) display altered pain thresholds.^38,49^ Although interesting, none of the studies to date conclusively demonstrate that the effects observed are because of MC ablation, rather than attributable to off target or compensatory effects.

Here we set out to test the role of MC in inflammatory pain using a novel tool which allows the specific ablation of these cells in an inducible manner by administration of diphtheria toxin (DTTx). In this model, DTTx treatment led to depletion of MC in the skin of Mcpt5-iDTR mice. Ablation of skin MC had no effect on mechanical hypersensitivity triggered by local injection of NGF. Furthermore, our experiments also demonstrate that loss of MC function has no effect in a long lasting acute model of inflammatory pain. Taken together, our results suggest that MC play, if any, only a very minor role in inflammatory pain.

## 2. Methods

### 2.1. Transgenic animal model

**Figure 1.**
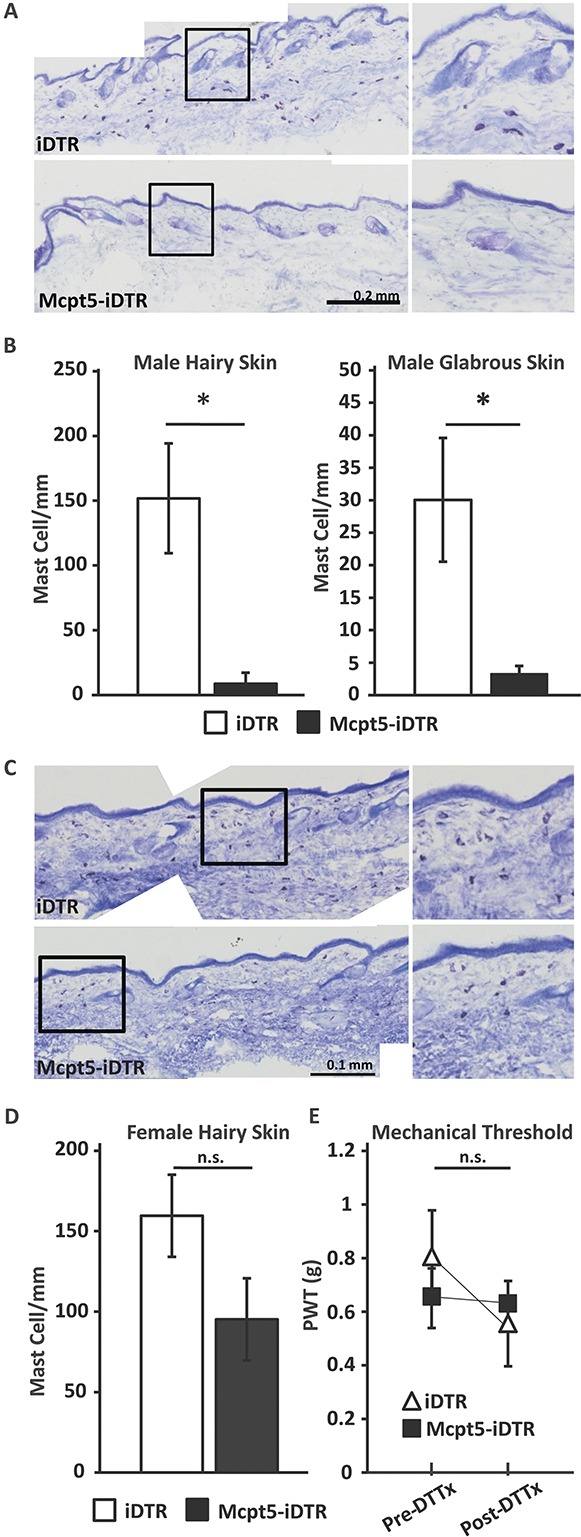
Depletion of mast cells (MC) in the Mcpt5-iDTR mouse model. (A and C) Representative pictures of hairy skin (dorsal paw) from Mcpt5-iDTR and control animals (iDTR) stained with toluidine blue, after treatment with diphtheria toxin (DTTx). Quantification shows that in males (B) MC were almost entirely depleted in both hairy and glabrous skin (*P ≤ 0.05). By contrast, Mcpt5-iDTR females (C and D) only showed a mild reduction in the number of MC in comparison with their littermate controls (iDTR) (*P* > 0.05) (E) Behavioural evaluation demonstrates no difference in mechanical withdrawal threshold between groups before or after DTTx treatment (main effect of treatment: F (1, 11) = 0.49, *P* > 0.05; main effect of time: F (1, 11) = 1.38, *P* > 0.05). Data are means ± SEM. n = 3 to 4 per group, 1-tailed Mann–Whitney test (A and B); n = 9 (iDTR) and 6 (Mcpt5-iDTR); 2-way repeated measures ANOVA (E).

Mcpt5-iDTR mice^26^ were obtained from the laboratory of Axel Roers and maintained homozygous for iDTR and hemizygous for Mcpt5-Cre on a C57BL/6J background. After initial skin mast cell depletion studies (Fig. 1A–D), all experiments were conducted in male mice only. The colony was maintained and genotyped by an independent experimenter, to ensure effective blinding during any behavioural testing.

### 2.2. Diphtheria toxin dosing

All mice were 3 to 9 months of age when DTTx treatment commenced. Mcpt5-iDTR (Mcpt5-Cre; iDTR^*flox/flox*^) and control littermates (iDTR^*flox/flox*^) were dosed with an i.p. injection of DTTx (25 ng/g; Sigma Aldrich) once a week over 4 consecutive weeks. Before the first injection, animals were injected with H1-antagonist pyrilamine (5 μg/g; Pyrilamine Maleate Salt, Sigma Aldrich) to avoid toxicity because of mast cell degranulation. All experiments were performed in accordance with the UK Animals (Scientific Procedures) Act 1986 and Local Ethical Committee approval.

### 2.3. Toluidine blue staining

Mice were deeply anesthetized and transcardially perfused with 4% PFA in PBS. Plantar skin was collected and postfixed for 1 hour at room temperature. Following fixation, tissue was immersed in 30% sucrose/PBS (overnight) and then mounted in OCT. Serial sagittal sections (10 μm thick) were cut with a microtome and immediately collected onto slides (Superfrost Plus–VWR) coated with 2% gelatine. Slides were rinsed in water, dipped in 0.5% toluidine blue solution (pH 4) for 2 minutes, washed with water, and mounted with DPX. Mosaics of single plane images were captured on using Axiovision LE Software, Axioskop microscope (Zeiss, Germany) with a 20 × 1.3 NA objective. Images (at least 4 mosaics per animal) were analysed by counting positive cells and normalised by the length of the skin sample.

### 2.4. Inflammatory pain and nerve growth factor sensitisation models

To model inflammatory pain, 20 μL complete Freund's adjuvant (CFA) (Sigma Aldrich) was injected into the intraplantar area of the left hind paw. Nerve growth factor was injected using the same method, at the concentration of 500 ng, dissolved in saline—20 µL final volume per animal.

### 2.5. Behavioural testing

In all behaviour paradigms, male adult animals (3-9 months) were used. Weights were monitored and annotated periodically. All the experiments were performed by an experimenter blind to genotype.

### 2.6. Mechanical withdrawal threshold

Mice were placed in a Perspex chamber on a wire mesh floor and allowed to acclimatise for at least 30 minutes. Withdrawal thresholds were determined using the up and down method,^15^ with a range of von Frey hair forces (0.04-2 g; Touch Test, North Coast Medical, Inc.). Calibrated hairs were applied to the plantar surface of the hind paw so the fibre would bend for approximately 2 seconds or until the animal withdrew its paw. A 50% paw-withdrawal threshold was calculated as previously described.^15^

### 2.7. Randall–Selitto (paw pressure)

Noxious mechanical threshold was evaluated using mechanical pressure stimulation based on the Randall–Selitto principle.^66^ In brief, animals were lightly restrained, and their hind paw was placed on the Analgesy-Meter apparatus (7200; Ugo Baseline). A probe with an increasing force was placed on the dorsal surface of the hind paw and the nociceptive threshold recorded as the force at which the animal responded by paw withdrawal. A maximum of 120 g pressure was applied to prevent any tissue damage.

### 2.8. Thermal withdrawal threshold

Thermal threshold of the hind paw was determined using an incremental hot/cold 20 cm diameter plate (Ugo Baseline) at a constant set temperature (51°C and 10°C, for hot and cold, respectively). For the noxious hot threshold, animals were gently placed on the plate, surrounded by a transparent acrylic cylinder and timed until they flinched, licked, shook the paw, or jumped. A maximum latency of 30 seconds was set to prevent blisters or other damage to the plantar skin. For the cold threshold, animals were gently restrained and their paw was tested by placing the plantar surface on the plate. The time to withdraw from the cold surface was recorded. In this test, a maximum latency of 20 seconds was allowed to prevent any tissue damage. In both tests, responses were recorded to a precision of 0.1 second.

### 2.9. In vivo diphtheria toxin toxicity evaluation

C57/BL6J male mice (3-6 months old) were dosed with an i.p. injection of DTTx (25 ng/g; Sigma Aldrich) or vehicle (0.9% saline) once a week over 4 consecutive weeks. Animals were then tested on different behaviour paradigms, as described above, 24 hours after each injection. All the experiments were performed by an experimenter blind to treatment.

### 2.10. Purified neuronal culture and in vitro Ca^++^ imaging

C57/BL6J adult mice were deeply anesthetized and transcardially perfused with 1x PBS. Dorsal root ganglia (DRGs) were collected and dissociated by enzymatic digestion, followed by gentle mechanical dissociation.^77^ Cell suspension was exposed to biotinylated nonneuronal antibody cocktail (Miltenyi MACS Neuron Isolation Kit), followed by antibiotin microbeads (Miltenyi MACS Neuron Isolation Kit). Cells were then run through a LD exclusion column and placed in a QuadroMACS separator (Miltenyi Biotech), so only neuronal cells were eluted (>95% pure neuronal cells generated).^77^ Neurons were then plated on matrigel coated coverslips and cultured for 48 hours in F12 medium (5% CO_2_, 95% O_2,_ at 37°C).

Following baseline measurements (3 minutes), neurons were exposed to compound 48/80 at 2 different concentrations, 10 and 100 μg/mL, and imaged for 3 minutes after each exposure. Cells were washed twice with Ca^++^ buffer in between treatments. Regions of interest were selected around cells and the ratio of the fluorescence intensity at 340/380 nm excitation was calculated. This fluorescence intensity ratio was normalised to the baseline ratio. The percentage of responding cells, after an application of Ca^++^ buffer and/or compound 48/80 at the 2 concentrations, was determined visually from ratiometric traces.

### 2.11. In vitro stimulation of mast cells

Bone marrow-derived MC(BMMC) were sensitized with monoclonal anti-Dinitrophenyl (DNP) immunoglobulinE, (clone SPE-7, Sigma) at 1 μg/mL overnight. The following day, BMMC were stimulated with DNP (50 ng/mL) alone or together with various agonists (UDP-glucose [1 μM], NGF [10 ng/mL]). After a 4 hours stimulation period, cell culture supernatants were collected, and cells were lysed for mRNA analysis.

### 2.12. Enzyme-linked immunospecific assay

Tumor necrosis factor**-**α (TNFα) cytokine production was measured in cell culture supernatants by standard enzyme-linked immunospecific assay (ELISA) using the mouse TNF-α DuoSet kit (R&D Systems), according to the manufacturer's instructions, and read on an ELISA plate reader (Molecular Devices) set at 450 nm.

### 2.13. Quantitative real-time polymerase chain reaction

Bone marrow-derived MC RNA was prepared with the RNeasy Micro kit (Qiagen) and total RNA (500 ng) was converted to cDNA using the superScript III Reverse Transcriptase kit (Thermo Fisher Scientific). Rat skin biopsies (glabrous skin) and rat DRGs (used as positive control) were collected and immediately processed using the same method as described above. Archival cDNA extracted from human skin punch biopsies was used to study the presence of NGF receptors in human skin. Quantitative real-time polymerase chain reaction (qRT-PCR) was performed in duplicate with a SYBR green master mix (Roche Diagnostics Limited) and the appropriate gene primers (Sigma) see below. ΔCts were calculated in relation to a house keeper gene (GAPDH). Reactions were run on a Roche Lightcycler 480 PCR machine, and results analysed by the standard ΔΔCt method. All primers were checked for their efficiency and specificity.

FCεR1_F: TGTGTACTTGAATGTAACGCAAGA; FCεR1_R: TGGACTAAGACCATGTCAGCA.

TrkA_F: GAAGAATGTGACGTGCTGGG; TrkA_R: GAAGGAGACGCTGACTTGGA.

p75_F: CCGCTGACAACCTCATTCC; p75_R: GGCTGTTGGCTCCTTGTTTATTT.

Gapdh_F: GGTCCCAGCTTAGGTTCATCA; GAPDH_R: CCAATACGGCCAAATCCGTTC.

TrkA_F (Human): CAGGACTTCCAGCGTGAG; TrkA_R (Human): CGGAGGAAGCGGTTGAG.

p75_F (Human): CTGTGGTTGTGGGCCTTGT; p75_R (Human): TGGAGTTTTTCTCCCTCTGGTG.

Gapdh_F (Human): GAAGGTGAAGGTCGGAGTCAAC; GAPDH_R (Human): CAGAGTTAAAAGCAGCCCTGGT.

### 2.14. Statistical analysis

All data are expressed as mean ± SEM. For all sets of data, normality of variance was assessed by a Shapiro–Wilk test. If samples showed a normal distribution, parametric tests were applied; otherwise, nonparametric tests were used. Statistical analyses were performed using GraphPad Prism Software.

## 3. Results

We obtained mice expressing a MC protease-Cre fusion gene (Mcpt5-Cre)^26^ which had been crossed with ROSA-floxed-STOP-iDTR mice that contain the sequence of the human DTTx receptor (DTR) in every cell.^11^ The resulting Mctp5Cre-iDTR mice express DTR only in MC, where the Cre can excise the stop signal. Administration of DTTx then leads to the conditional ablation of MC while sparing all other immune cell types.^11,26,68^ During their first characterisation, Mcpt5-iDTR mice were shown to be almost completely depleted of MC in the peritoneal cavity, ear, and back skin after DTTx administration.^26^ To further characterise and determine whether MC could also be ablated in dorsal and plantar paw skin (hairy and glabrous skin, respectively), we carried out immunohistochemistry after DTTx treatment (4 i.p. injections over 4 weeks) in both male and female Mcpt5-iDTR transgenic mice. Our histology showed a considerable and significant reduction (∼95%) in the number of MC in male Mcpt5-iDTR mice in relation to their littermate controls (Fig. 1A), demonstrating a successful depletion of MC in both hairy and glabrous paw skin in transgenic males (Fig. 1B). Interestingly, in female Mcpt5-iDTR mice the number of MC only dropped by approximately 40%, a reduction which was not significant in comparison with Cre negative iDTR littermates (Fig. 1C, D).

In addition to histological characterisation, we also investigated whether loss of MC in plantar skin causes any behavioural changes in the Mcpt5-iDTR male mouse model. We found no alterations in mechanical pain threshold before or after DTTx (paw-withdrawal responses remained unaffected in a von Frey test; Fig. 1E). Furthermore, to rule out any possible effects DTTx could have on sensory neurons in vivo, we tested wildtype mice which were submitted to DTTx treatment, on different behavioural paradigms. Our results show that DTTx treatment has no acute (3 hours after first injection), long term (24 hours after first injection), or chronic effect (4 injections over 4 weeks) on mechanical (von Frey and Randall–Selitto) or thermal (hot and cold) thresholds compared with the control (vehicle) group (Supp. Fig. 1, available online as supplemental digital content at http://links.lww.com/PAIN/A405). Importantly, mechanical and thermal thresholds remain unchanged pretreatment and posttreatment (Supp. Fig. 1A–D, available online as supplemental digital content at http://links.lww.com/PAIN/A405), except on the cold plate, where both groups displayed some learning behaviour (Supp. Fig. 1D, available online as supplemental digital content at http://links.lww.com/PAIN/A405). Together these results indicate that the Mcpt5-iDTR male model is a good system to study the involvement of MC in peripheral sensitisation and inflammatory pain.

To date, most model systems used to study MC function in pain present a few drawbacks. For instance, the use of compound 48/80, known for degranulating and depleting MC, has been found to have off target effects, also acting directly on other cell types.^17,51,70^ To further validate the uniqueness of the Mcpt5-iDTR model to study MC in pain, and to investigate whether compound 48/80 has a direct effect in sensory neurons, we used Ca^++^ imaging to monitor the neuronal response and excitability on exposure to different concentrations of compound 48/80. Notably, for these experiments, purified DRG neurons were used, allowing over 95% cell purity^77^ and therefore, excluding any response driven by other nonneuronal cell types. We found that following baseline recording, at a lower concentration of compound 48/80 (10 μg/mL), approximately 20% of neurons show an increased excitability almost immediately after the exposure to compound 48/80 and lasting up to 3 minutes of recording (Supp. Fig. 2A, B, available online as supplemental digital content at http://links.lww.com/PAIN/A405). Remarkably, when the concentration of compound 48/80 was increased (100 μg/mL), almost 70% of the neurons responded to the treatment (Supp. Fig. 2A, B, available online as supplemental digital content at http://links.lww.com/PAIN/A405). It cannot be ruled out that at this high concentration compound 48/80 may even be toxic to neurons, as intracellular Ca^++^ accumulation remains constant after several minutes after the application of the drug. Nevertheless, our data clearly demonstrate that DRG neurons are capable of responding to compound 48/80, at a dose known to cause MC degranulation (10 μg/ml)^13,33,58,72,75,78^ and independent of the presence of any other cell type. These findings further demonstrate the unspecificity of compound 48/80 and emphasise the importance of developing new models, such as the Mcpt5-iDTR transgenic model, to study MC in context of pain.

**Figure 2.**
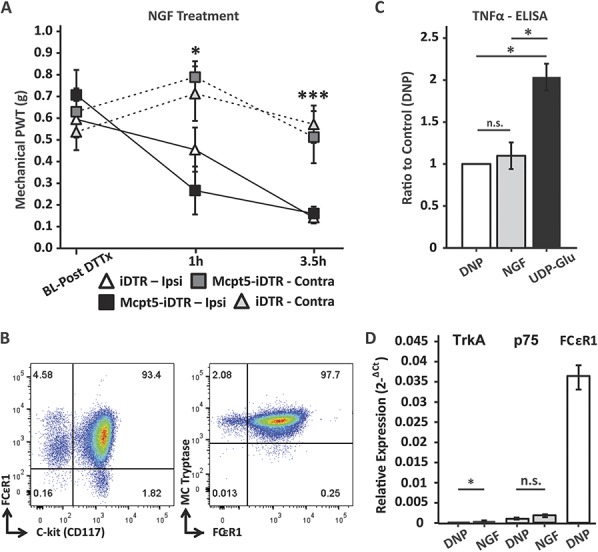
Nerve growth factor (NGF) treatment has no effect on mast cells (MC). (A) Intraplantar injection of NGF shows a similar decrease in mechanical threshold in both groups of animals, 1 hour after injection and 3.5 hours after the injection (F_(2,36)_ = 23.40; *P* < 0.001). Data from baseline and contralateral paw demonstrates no underlying differences between MC depleted animals and control littermates (F_(1,18)_ = 0.05; *P* > 0.05). (B) FACS dot plots showing gating strategy for bone-derived MC. (C) Graph showing the quantification of tumor necrosis factor-α release (enzyme-linked immunospecific assay [ELISA]) from Bone marrow-derived MC (BMMC) after treatment with NGF, in comparison with basal levels (dinitrophenyl [DNP]) and treatment with UDP-Glucose (positive control). (D) qRT-PCR for NGF receptors on RNA extracted from BMMC (n = 3 experiments), before (DNP) and after treatment with NGF (TrkA: black bars; p75: gray bars), in comparison with basal levels of FCεR1 (white bar). Data are means ± SEM. n = 12 (iDTR) and 8 (Mcpt5-iDTR); 2-way ANOVA repeated measures (A).

Nerve growth factor is well known for being secreted during injury and inflammation, leading to a rapid sensitisation of peripheral nociceptors.^41,52,62^ Indeed, blockade of NGF signalling has been shown to attenuate allodynia in different models of persistent pain, and more recently an anti-NGF antibody has reached phase II and III clinical trials, proving to be a promising target to alleviate chronic pain.^27,28,44,73^ Early studies also suggested that NGF is capable of both activation and proliferation of MC in peripheral tissues.^3,4,45,63^ We, therefore, used the NGF sensitisation model^6,22,42,43,55,80^ to investigate the role of MC in acute inflammatory pain and nociceptor sensitisation. As expected, after intraplantar NGF injection, we observed increased mechanical hypersensitivity in the injected paw of control animals, as demonstrated by decreased paw-withdrawal thresholds in the von Frey test (Fig. 2A). Yet, both control and Mcpt5-iDTR groups showed the same level of sensitisation triggered by NGF, suggesting that MC do not potentiate its pronociceptive effects.

Given these unexpected results, and to rule out any possible compensatory factors, we went on to investigate the direct effect of NGF on isolated BMMC in vitro. To check the quality and purity of BMMC, we first analysed our cultures by flow cytometry. We found that 98% of our cells expressed c-kit, FCεR1, and mast cell tryptase, confirming successful differentiation of bone marrow cells into MC after 4 to 6 weeks in culture (Fig. 2B). To test the effect of NGF on MC activation, we sensitised the cells using DNP and immunoglobulin E (IgE) and treated them with NGF (4 hours, 10 ng/mL). UDP-Glucose was used as positive control, as it is known to stimulate and induce production of TNFα by MC.^31,36^ Nerve growth factor did not induce increased secretion of TNFα when compared with DNP alone, as measured by ELISA (Fig. 2C). To clarify these findings, we checked the expression levels of the 2 NGF receptors—TrkA and p75—in BMMC via qRT-PCR. Our data demonstrate that basal levels of both NGF receptors in MC were negligible (Fig. 2D). After treatment with NGF, there was a very moderate increase in the levels of TrkA and p75 mRNA in MC (Fig. 2D). Nevertheless, levels of NGF receptors were negligible when compared with basal levels of FCεR1 (Fig. 2D). Similarly, levels of NGF receptors were also negligible in peritoneal MC (data not shown). Overall, our results demonstrate that NGF treatment has no effect on MC as these cells do not express NGF receptors.

To further explore the link between NGF and MC, and the relevance of our findings to other systems, we went on to investigate whether NGF receptors are expressed in rat and human MC. Our qRT-PCR results indicate that, similarly to mice, NGF receptors are not present in the MC of rats or humans, as levels of TrkA and p75 were negligible in the skin samples analysed (Supp. Fig. 3A, B, available online as supplemental digital content at http://links.lww.com/PAIN/A405). These results reinforce our previous findings indicating that MC do not express NGF receptors.

**Figure 3.**
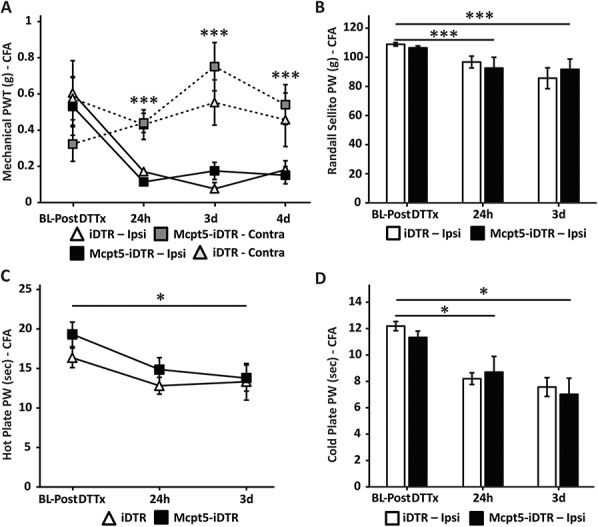
Loss of mast cells (MC) does not reduce peripheral sensitisation during inflammation. (A) Paw withdrawal on the von Frey test showed a decreased mechanical threshold in both groups of animals after intraplantar complete Freund's adjuvant (CFA) injection (F_(9,66)_ = 2.94; *P* ≤ 0.005), 24 hours, 72 hours and 4 days after injection (F_(3,66)_ = 12.30; *P* < 0.001). Baseline and contralateral paw-withdrawal thresholds demonstrated no underlying differences between groups (F_(3,22)_ = 2.45; *P* > 0.05). (B) Paw withdrawal on the Randall–Selitto test further demonstrated a sensitisation to a noxious mechanical stimuli after CFA injection (F_(6,52)_ = 6.10; *P* < 0.001), both 24 and 72 hours after injection (F_(2,52)_ = 25.41; *P* < 0.001). (C) CFA treatment equally changes thermal hypersensitivity on the hot plate (F_(2,26)_ = 4.47, *P* < 0.05) and cold plate (F_(2,26)_ = 25.41; *P* < 0.001) (D). Data are means ± SEM. n = 9 (iDTR) and 6 (Mcpt5-iDTR); 2-Way repeated measures ANOVA.

**Figure 4.**
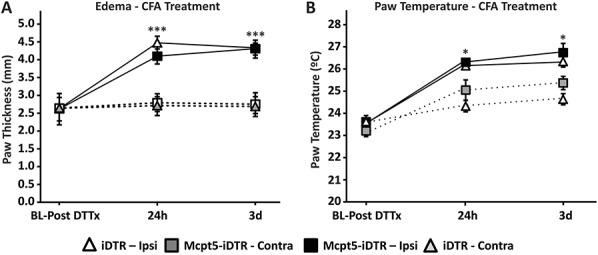
Depletion of mast cells has no impact on gross physiological changes triggered by inflammation. (A) Measuring of edema after complete Freund's adjuvant (CFA) injection shows a significant swelling in comparison with baseline (F_(9,78)_ = 75.47; *P* < 0.001), but no difference between groups (F_(1,13)_ = 1.69; *P* > 0.05). (B) Similarly, paw temperature in the injected area increased after inflammation (F_(2,52)_ = 74.22; *P* < 0.001), but was very similar in iDTR and Mcpt5-iDTR animals (F_(26,52)_ = 0.56; *P* > 0.05). Data are means ± SEM. n = 9 (iDTR) and 6 (Mcpt5-iDTR); 2-way repeated measures ANOVA.

To rule out the possibility that our stimulus (NGF) might have been too specific or mild to lead to MC recruitment and activation, we set out to study a much stronger proinflammatory insult. For these experiments we used the well-characterised, CFA model, which induces chronic inflammation and hypersensitivity at the injection site, as well as the recruitment and activation of MC.^14,50,57,59,67,82^ Our experiments demonstrate that depletion of MC, using the Mcpt5-iDTR model, had no impact on mechanical and thermal hypersensitivity thresholds 24 hours after intraplantar injection of CFA. No significant differences emerged between MC depleted mice and their littermates on von Frey, Randall–Selitto, hot or cold plate tests (Fig. 3A–D). Crucially, when evaluating later stages after the acute phase (3 and 4 days after CFA injection), we still did not observe any change in mechanical or thermal hyperalgesia between the 2 groups of mice (Fig. 3A–D). Furthermore, edema (paw thickness) and temperature triggered by CFA inflammation was consistent between the groups (Fig. 4A, B), both in acute (24 hours) and longer-term inflammation (3 days). Together, our data indicate that MC contribute little to the sensitisation of peripheral nociceptors during CFA-mediated inflammation.

## 4. Discussion

In this study, we have analysed the role of MC in the sensitisation of peripheral nociceptors during acute inflammation. Our evidence indicates that MC appear to not be essential for this process; in the Mcpt5-iDTR mouse model, where almost complete depletion of MC in glabrous skin was achieved, animals still displayed normal levels of mechanical hypersensitisation after local injection of NGF. This was further supported by our in vitro studies, demonstrating that NGF alone had no effect on the level of TNFα secreted by MC. Similarly, when challenged with a strong inflammatory stimulus (CFA), Mcpt5-iDTR mice still presented the same levels of mechanical and thermal hyperalgesia as their control littermates, both in acute and longer-term phases (3-4 days) of inflammation. Sensitisation of peripheral nociceptors during inflammation is, therefore, likely to be mediated and potentiated by immune cells other than MC.

Evidence for the contribution of MC to acute inflammatory sensitisation of nociceptors is not strong. Previous studies have implied that loss of MC function could be implicated in pain, and induction of this process could, therefore, represent a potential target for alleviating acute peripheral neuronal sensitisation^23,25,38,47,49,69,81,85^. However, most of these studies suffered from many confounding factors due to their experimental design. For instance, compound 48/80, which is broadly used to acutely degranulate and deplete MC, has been found to have a profound impact on other immune cells, including neutrophils and eosinophils^17,51^ and directly affects sensory neuron excitability, as shown in this study, and previously suggested by Schemann et al.^70^ Furthermore, interpretation from studies using more refined techniques such as MC transgenic lines have equally proven to be ambiguous, in particular those using c-kit transgenics. Recent findings have shown that constitutive disruption of c-kit signalling has significant consequences for the function and number of many immune cell types other than MC, such as erythrocytes and neutrophils.^30,39^ Crucially, beyond the immune system, it has been shown that c-kit is expressed in spinal cord neurons and nociceptors,^54,74^ a result which is further supported by recent RNA sequencing data.^77,79^

To overcome these limitations, in our study we used an established transgenic line (Mcpt5-iDTR), where MC deficiency can be induced by administration of DTTx^11,26^ and has no impact on other immune cell populations.^26,39^ We have shown almost complete depletion of MC in the paw skin of this Mcpt5-iDTR model, and the reduction in the number of cells was comparable in size with that reported in other tissues in this same transgenic system.^26^

We also found sex differences when attempting to deplete MC, with MC counts in female mice remaining almost unaffected by DTTx treatment. It is likely that this observation is because of the role that female hormones play in MC behaviour, affecting their number and degranulation, as previously reported.^9,35,84^ These findings mean that we cannot comment on the role of MC in female mice because of the specifics of our experimental design. More importantly, they also imply that particular care has to be taken when designing future investigations into the role of MC in pain, particularly in females.

Acute NGF response, MC and hypersensitisation: a cross-talk pathway? NGF has a well-established role in the adult nociceptive system, mediating and modulating pain, as well as causing changes in gene expression, particularly in persistent pain states.^41,52,65^ Acute NGF treatment leads to mechanical and thermal hyperalgesia,^6,22,42,43,55,80^ an effect that was believed to be directly linked to MC activation.^3,4,45,47,63,69,81^ Surprisingly, our results revealed that depletion of MC in vivo does not reduce acute NGF-induced peripheral sensitisation. In addition, our in vitro experiments further supplemented these findings and demonstrated that NGF does not activate MC. We were also able to show that MC express neither of the 2 NGF receptors, TrkA or p75, explaining their lack of response on exposure to NGF. Importantly, our results are in line with recent RNA-seq data which show that bone marrow-derived, peritoneal and intestinal MC have none or negligible levels of TrkA and p75.^12,18^ In addition, we also show similar results in samples from rat and human skin, with our data once more replicating previously published RNA-seq data.^21^ Although a dilution effect cannot be excluded when studying MC in skin, the overwhelming majority of recent expression data support our conclusion that MC play an inconsequential role in NGF-mediated nociceptor sensitisation, primarily because they lack NGF receptors. Naturally, it cannot be ruled out that more chronic inflammatory conditions eventually upregulate NGF receptors on MC, rendering them directly sensitive to this particular pain mediator. Or indeed, it could be that long-term exposure to NGF has an indirect effect on MC function, increasing other important inflammatory mediators in these cells that then go on to impact sensory neurons or other immune cell types.

Do MC play a role in the inflammatory response? Our results demonstrate that MC have no evident immediate role in NGF or CFA triggered sensitisation, including its more persistent phases. These results are supported by a recent study which demonstrates that CFA-induction and maintenance of mechanical and thermal hyperalgesia are primarily dependent on specific populations of myeloid cells, particularly macrophages.^32^ These findings imply that pursuing MC as a target to alleviate mechanical and thermal allodynia, as well as to attenuate edema and other physiological changes that arise immediately after inflammation, might not be the most appropriate approach to tackle pain during inflammation. Although we show no obvious function for MC at early to mid-term stages of sensitisation, we speculate that during long-term inflammatory conditions MC may get primed to potentiate inflammatory responses, and therefore, may have a potential impact on nociceptors in a chronic pain scenario. According to this view, sensitisation would occur as a result of more complex transcriptional and molecular alterations. Future studies exploring whether MC can directly sensitise afferents during long periods of inflammation will be necessary to fully understand their role–if any–in more persistent pain conditions.

## Conflict of interest statement

The authors have no conflict of interest to declare.

## Supplementary Material

SUPPLEMENTARY MATERIAL

## Appendix A. Supplemental Digital Content

Supplemental Digital Content associated with this article can be found online at http://links.lww.com/PAIN/A405.
