# Supplementary figures and images for: Peripheral inflammatory pain sensitisation is independent of mast cell activation in male mice
Source: Pain. 2017 Apr 5;158(7):1314–22. doi: 10.1097/j.pain.0000000000000917 (PMC5472008; doi:10.1097/j.pain.0000000000000917)

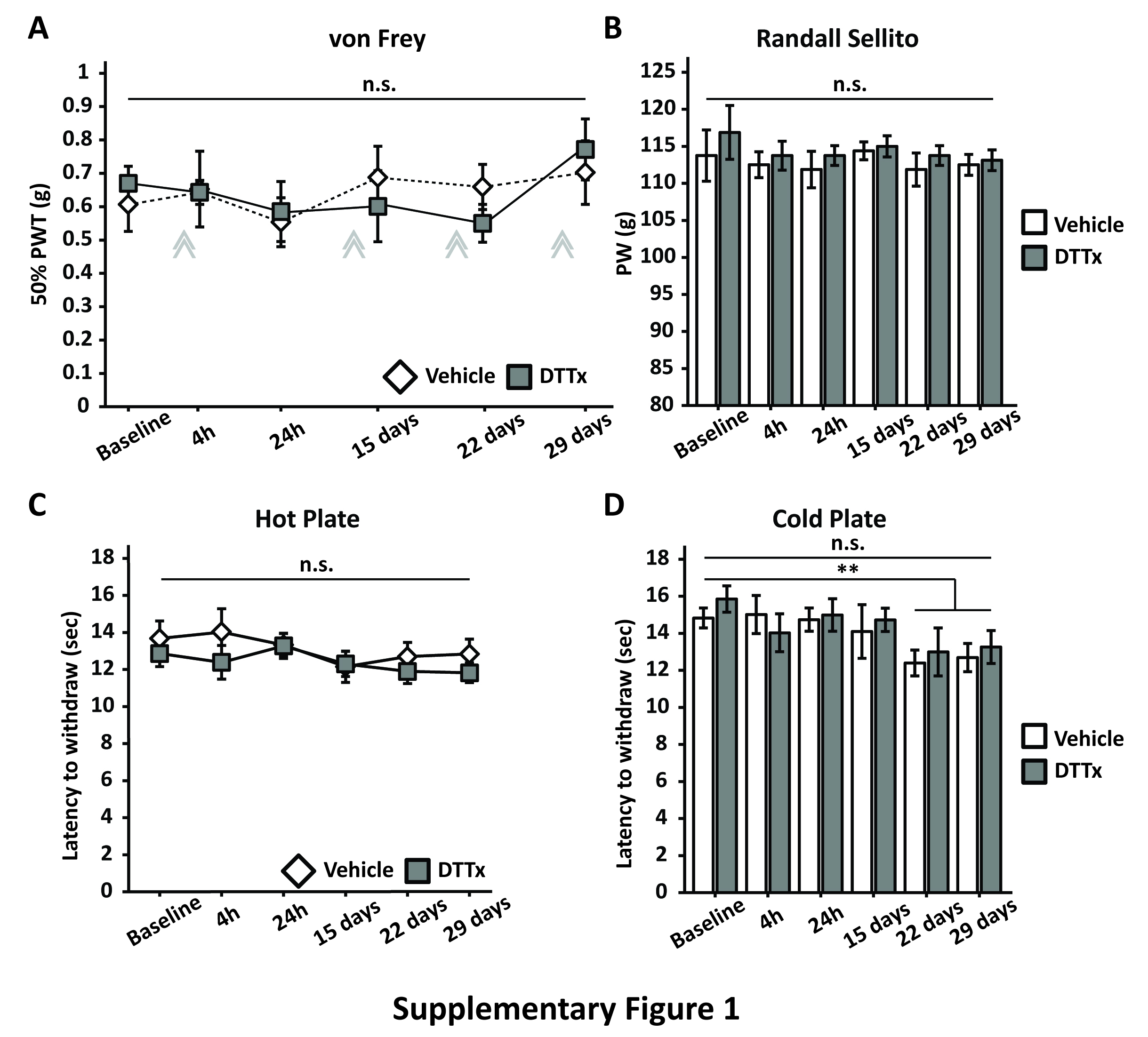

Supplement: SUPPLEMENTARY MATERIAL [file jop-158-1314-s001.jpg]

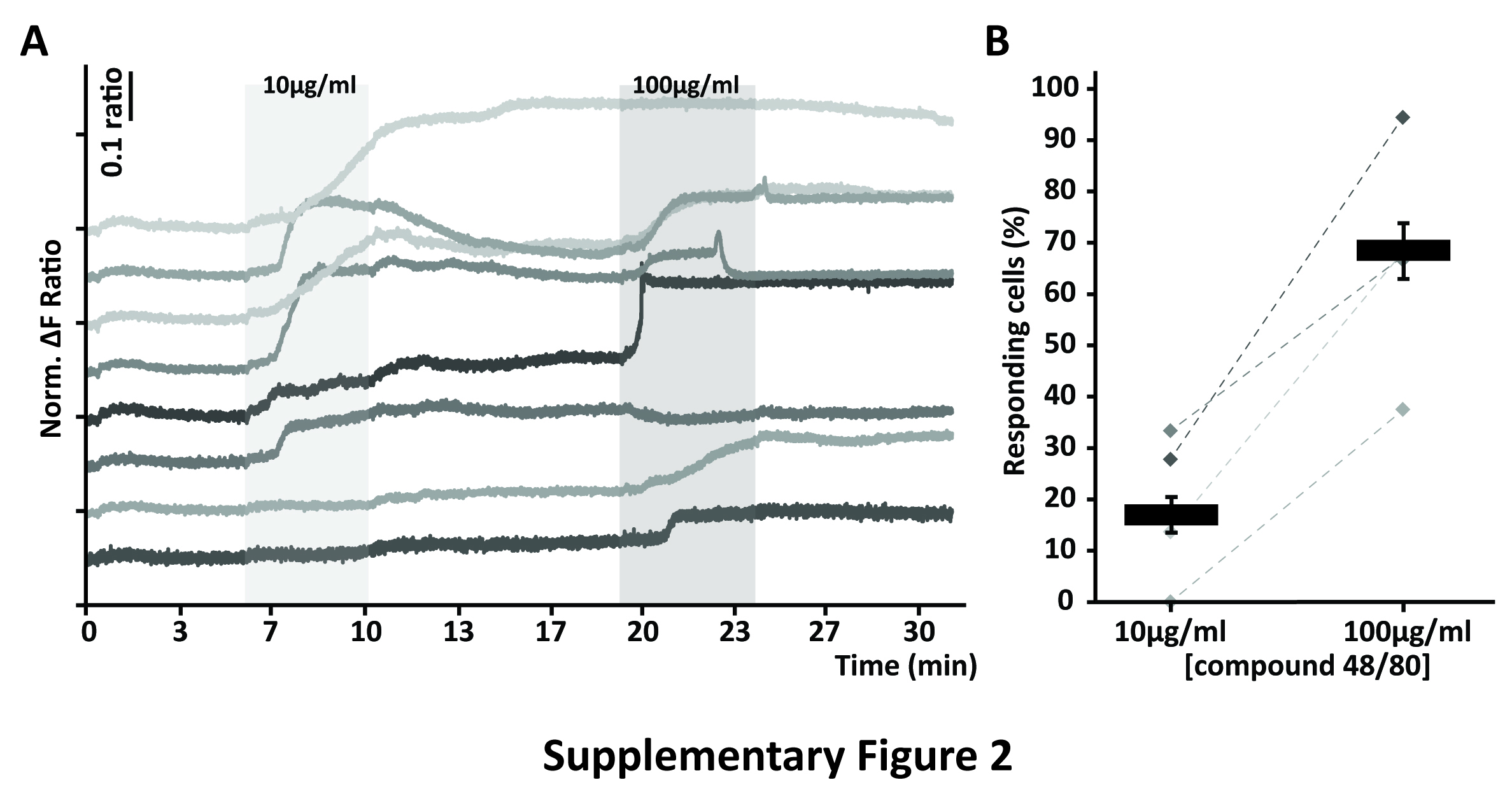

Supplement: SUPPLEMENTARY MATERIAL [file jop-158-1314-s002.jpg]

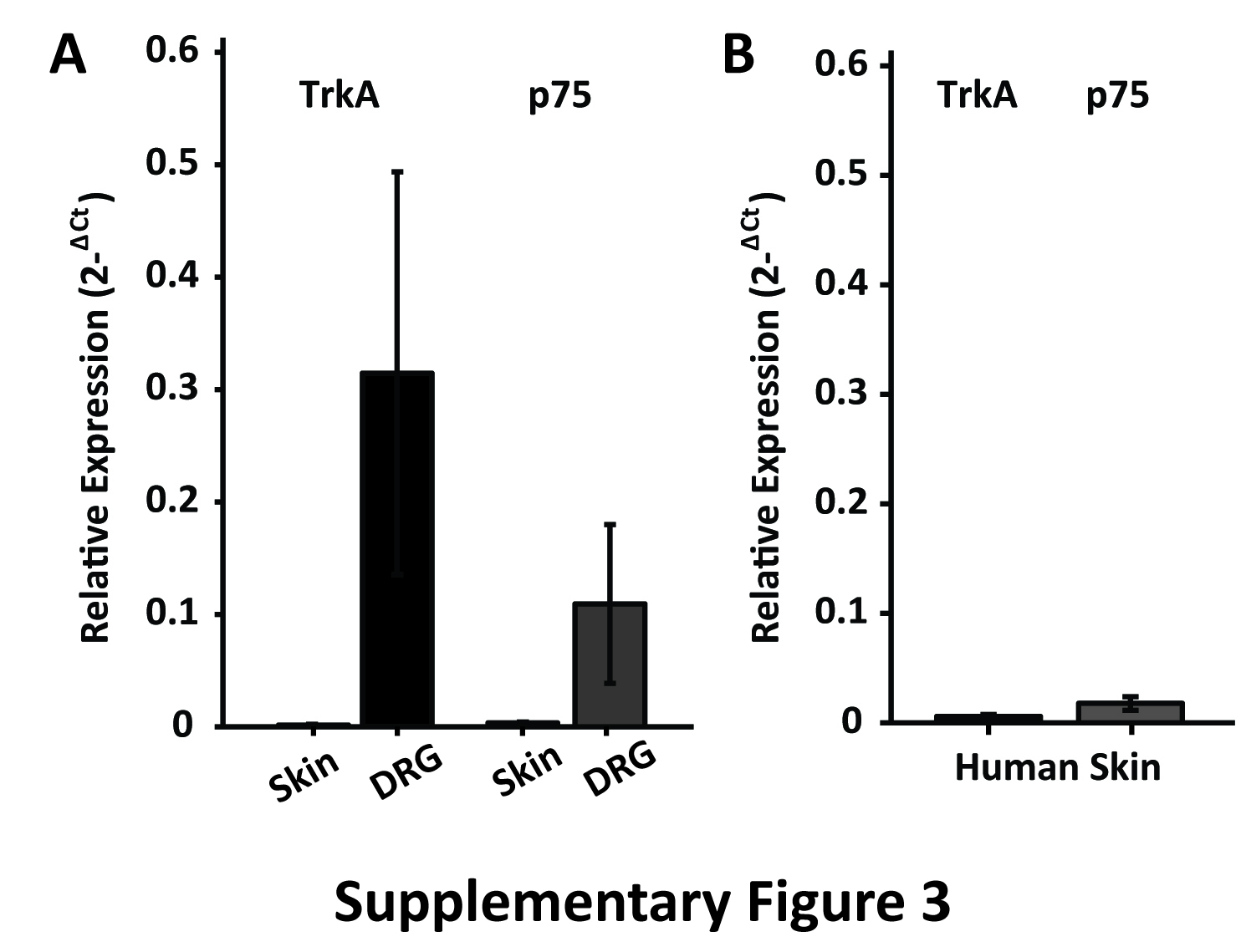

Supplement: SUPPLEMENTARY MATERIAL [file jop-158-1314-s003.jpg]
