# Supplementary material for: Peripheral inflammatory pain sensitisation is independent of mast cell activation in male mice
Source: Pain. 2017 Apr 5;158(7):1314–22. doi: 10.1097/j.pain.0000000000000917 (PMC5472008; doi:10.1097/j.pain.0000000000000917)
Supplement: SUPPLEMENTARY MATERIAL [file jop-158-1314-s004.docx]

**Supplementary Figure Legends**

Supplementary Figure 1: DTTx treatment does not alter mechanical and thermal sensitivity in mice. Animals received four injections of DTTx or vehicle – day 1, 14, 21 and 28 – and were tested periodically. A) Paw withdrawal on the von Frey test showed no difference in mechanical threshold between groups (vehicle and DTTx) (F(1,14)=0.12P>0.05). Time course behavioural analyses showed no change in mechanical sensitivity both acutely (4h after injection) and chronically (24h, 15 days, 22 days and 29 days) after injections (F(5,70)=1.50; P>0.05). DTTx treated animals presented the same behaviour patterns as control animals in the Randall-Selitto test (F(1,14)=1; P>0.05), hot plate (F(1,14)=0.85; P>0.05) and cold plate (F(1,14)=0.85; P>0.05), B, C & D, respectively. Interestingly, both group showed some learning behaviour at later points of the cold plate test (D). Data are means ± SEM. Grey chevrons represent 1st, 2nd, 3rd and 4th injections of either vehicle or DTTx. n= 8/group; two-way repeated measures ANOVA.

Supplementary Figure 2: Compound 48/80 acts directly on DRG neurons. A) Representative traces of cells responding to the application of compound 48/80 at 10μg/ml and 100μg/ml concentrations (shaded areas). Data are displayed as a normalized ratio of 340/380 fluorescence (ΔF Ratio). B) Percentage of cells responding to the application of two concentrations of compound 48/80. Data are means ± SEM, n = 4 mice and 67 cells. Grey diamonds (B) represent the average response for each animal.

Supplementary Figure 3: Absence of TrkA and p75 expression in rat and human skin. A) qRT-PCR for NGF receptors on RNA extracted from the plantar skin of rats (n = 4) - TrkA: black bars; p75: grey bars – demonstrate absence of TrkA and p75 in the samples. Rat DRGs were used as a reference for the level of expression (n = 2). B) Similarly, human skin (n = 4) appears to be devoid of TrkA and p75, suggesting very low or no expression of NGF receptors in MC. Data are means ± SEM.
